# A Simple Assay to Assess *Salmonella* Typhimurium Impact on Performance and Immune Status of Growing Pigs after Different Inoculation Doses

**DOI:** 10.3390/microorganisms11020446

**Published:** 2023-02-10

**Authors:** Graziela Alves da Cunha Valini, Pedro Righetti Arnaut, Larissa Gonçalves Barbosa, Paulo Henrique Amadeu de Azevedo, Antonio Diego Brandão Melo, Danilo Alves Marçal, Paulo Henrique Reis Furtado Campos, Luciano Hauschild

**Affiliations:** 1 Department of Animal Science, Faculdade de Ciências Agrárias e Veterinárias, UNESP, Universidade Estadual Paulista “Júlio de Mesquita Filho”, Jaboticabal 14884-900, São Paulo, Brazil; 2 Department of Animal Science, Universidade Federal de Viçosa, Viçosa 36570-900, Minas Gerais, Brazil

**Keywords:** growth, immunologic response, sanitary challenge, swine

## Abstract

*Salmonella* Typhimurium is the most frequent serovar in pigs and causes infections in humans. However, the dosage used for experimentation is not well defined. The present study aimed to evaluate a dosage for oral inoculation with *Salmonella* Typhimurium to assess immunological and growth performance alterations in pigs. Gilts were randomly allocated into one of three experimental treatments: no *Salmonella* Typhimurium inoculation (Basal), or oral inoculation of 1 × 10^8^ or 1.5 × 10^8^ colony-forming units of *Salmonella* Typhimurium. Growth rate, rectal temperature, and fecal *Salmonella* shedding were recorded. Blood samples were taken. Inoculated pigs shed the bacteria for up to 7 days, but no differences were observed between the groups. No differences were observed in rectal temperature, body weight, or average daily feed intake. However, reductions in average daily gain (−17 and −22%) and feed efficiency (−14 and −20%) were observed in pigs inoculated with 1 × 10^8^ and 1.5 × 10^8^ colony-forming units, respectively. The hemoglobin and hematocrit concentrations increased in challenged pigs compared to Basal pigs. The oral dosage of 1.5 × 10^8^ colony-forming units of *Salmonella* Typhimurium is suitable for activating the immune system of pigs and assessing the impact of *Salmonella* on pig performance.

## 1. Introduction

The optimal growth performance of growing–finishing pigs can be affected by a wide range of environmental and sanitary factors. In commercial production systems, highly intensified production, the management conditions, and the assumed lower capacity of modern genotypes to adapt to environmental and sanitary challenges are likely to predispose pigs to recurrent immune responses [1,2].

The *Salmonella* enterica serovar Typhimurium (ST) has become the most common serovar in pig production worldwide [3], including in Europe, the United States, and Brazil [4,5,6]. Additionally, ST has been recognized as a foodborne disease in humans, as contaminated pork is an considerable source of *Salmonella* infections in humans [7]. Along with the potential for pork contamination, there are also health and production losses that ST infections can cause in pigs. For example, it can lead to increased drug use, increased time to market, and loss of premiums due to variability in carcass weight [2].

The ST infection can occur at any stage of pig production, but growing–finishing pigs are the main target of ST. It has a great capacity to persist in the environment for long periods [8], and in the gut and gut-associated lymphoid tissues [9], making pigs frequent ST carriers. The transmission occurs mainly via the fecal–oral route. The clinical signs in pigs infected with ST include lethargy, anorexia, and the development of enterocolitis, beginning with watery diarrhea (with or without blood) that lasts 3 to 7 days, followed by dehydration; however, mortality is usually low [10]. In addition, growth performance (reduced weight gain and feed gain ratio) is generally reduced in ST-infected pigs, in part resulting from the redistribution of nutrients [11] to support the synthesis of immunological molecules, maintenance of the intestinal barrier, and oxidative status.

However, studies using ST as a challenge model in growing pigs are scarce, and its deleterious effects depend on the inoculum dosage, strain virulence, and hosts’ health status. The dose of ST used in studies usually varies from 10^7^ to 10^9^ colony-forming units (CFU), as it was previously reported that growing–finishing phase pigs can shed high concentrations of ST in the environment (between 10^5^ and 10^8^ CFU/g of feces) [12,13]. However, such a range of doses results in different outcomes. Some authors reported models with mild infectious outcomes [14,15,16]; while other studies showed clinical responses [17,18], and even responses that overwhelmed treatment capacity [19].

Thus, before proposing strategies to mitigate the negative impact of ST, it is important to determine a suitable challenge model regarding their practicality, as well as inoculum dosage and immunological and physiological alterations without inducing a severe illness. Understanding the impact of the challenge on the immune system and performance responses may aid future researchers in choosing a more efficient attenuating strategy for pigs with ST. Therefore, this study aimed to determine the dosage for oral gavage inoculation with ST required to assess immunological, physiological, and growth performance alterations in pigs.

## 2. Materials and Methods

### 2.1. Animals, Housing and Management

All experimental procedures in this trial followed the guidelines of the Brazilian National Council of the Control of Animal Experimentation and were reviewed and approved by the Ethical Committee on Animal Use of São Paulo State University (protocol no. 4784/20).

The study was conducted at the Swine Research Facility of São Paulo State University (FCAV/UNESP, Jaboticabal, Brazil) in two similar open-sided nursery buildings. Before the arrival of the pigs, both buildings were cleaned and disinfected with iodine 2.6% iodophor (dilution 1:250, Biocid; Pfizer, Brooklyn, NY, USA) in accordance with the manufacturer’s instructions. Additionally, prior to the allocation of the animals (day −7), surface drag swabs were taken in both buildings to screen for *Salmonella* spp. The swabs were serially diluted in buffered peptone solution (1:10) until they reached a final concentration of 10^−6^. From each dilution, 0.1-mL was plated on brilliant green agar (CM0263; Oxoid, Basingstoke, Hampshire, England), and incubated at 37 °C for 24 h. No *Salmonella* spp. presence was detected in the facilities.

Thirty crossbred female pigs (72 days old; Pietran × (Large White × Landrace)) with an initial average body weight (BW) of 27.3 ± 5.1 kg were used in a 14day trial. The female pigs were obtained from a previously selected farm, free of *Salmonella* spp., with good biosecurity measures, and located near the municipality of Jaboticabal-SP.

The pigs were individually housed in suspended pens with fully slatted plastic floors (1.4 × 1.5 m). Each pen was equipped with a one-sided self-feeder and a nipple drinker that allowed ad libitum access to feed and water. Before the study started, rectal swabs were collected individually to verify that the pigs were free of detectable *Salmonella* spp. shedding. Rectal swabs were diluted, plated, and incubated using the same surface drag swab protocol. All pigs were negative for *Salmonella* spp.

Throughout the trial, all pigs were fed a mashed standard corn–soybean meal-based diet, formulated to meet the nutrient requirements according to the NRC [19]. The standard diet was formulated with 2548 kcal/kg of net energy and a standard ileal digestible lysine content of 9.9 g/kg. The standard ileal digestible methionine + cysteine, threonine, tryptophan, and valine contents expressed as a percentage of digestible lysine were 56, 59, 17 and 68%, respectively, according to the amino acid profile [20].

### 2.2. Experimental Design

On Day 0, pigs were randomly assigned to one of three treatments according to BW with ten replicates of one animal per treatment. Treatments consisted of no ST inoculation (Basal), 1 × 10^8^ CFU of ST inoculation, and 1.5 × 10^8^ CFU of ST inoculation.

The Basal group was inoculated by oral gavage with 5-mL of brain heart infusion (BHI, CM 1135; Oxoid, Thermo Fisher Scientific, Hampshire, England) broth solution without ST and kept in a separate facility to avoid cross-contamination. The ST-challenged groups received an oral inoculum with 5-mL of BHI broth solution containing 1 × 10^8^ CFU of ST or 1.5 × 10^8^ CFU of ST. The inoculum was administrated into the mouth of the animals using a 5-mL syringe coupled with an orogastric tube. The liquid was slowly pushed into the esophagus according to the pigs’ deglutition. The gilts were fasted for six hours and had no water consumption for 1-h prior to inoculation.

For inoculation, the oral dose of *Salmonella enterica* subsp. enterica serovar Typhimurium was prepared from a sample of *Salmonella* Typhimurium (RL0971/09) originally isolated from swine feces and naturally resistant to nalidixic acid (Nal+). This ST sample was obtained from the Laboratory of Ornitopathology of the Department of Veterinary Pathology (FCAV/UNESP, Jaboticabal-SP). The inoculum strain was tested for its antimicrobial sensitivity on Mueller–Hinton agar (CM0337; Oxoid, Basingstoke, Hampshire, UK), with the Kirby–Bauer method [21], using commercial discs impregnated with the following antibiotics: gentamicin, florfenicol, cefotaxime, cephalothin, ciprofloxacin, ceftriaxone, polymyxin B, tetracycline, streptomycin, nalidixic acid, ampicillin, chloramphenicol, sulfamethoxazole + trimethoprim, and novobiocin. The antibiogram test was also performed to evaluate if the *Salmonella* excreted by pigs, after inoculation, had the same pattern of sensitivity or resistance to the antibiotics tested with the strain used in the preparation of the inoculum.

The inoculum was prepared according to the recommendations of Wood et al. [22] and Oliveira et al. [23]. Briefly, the inoculum was prepared from a frozen culture inoculated in BHI overnight at 37 °C. On the following day, the bacterial culture was diluted in phosphate-buffered saline (to contain 10^8^ CFU) and incubated for 24h at 37 °C. Then, the inoculum concentration was confirmed by the drop-counting technique.

After inoculation, to mimic a commercial housing condition, and to assist in exacerbating the inflammatory response and maintaining the challenge as long as possible, no cleaning routine was adopted in the ST-challenged facility. On the other hand, the Basal group facility was cleaned twice a day, and it was washed with a bleach solution (1:10) once a week as part of the biosecurity protocol. During the experimental period, the team members were required to wear clean and disinfected clothing and footwear was cleaned with a bleach solution (1:10) when entering the facility.

### 2.3. Data and Sample Collection

Feed provided and feed wastage were collected and weighed daily to determine the average daily feed intake (ADFI). The BW was recorded weekly to estimate the average daily gain (ADG) and feed efficiency (F:G).

The rectal temperature (RT) and the fecal score (FS) were measured daily in all pigs from 0 to 7 days post inoculation (dpi). The RT was recorded using a digital thermometer (Accumed-Glicomed, Rio de Janeiro, Brazil), and the FS was attributed to each pig with the following scoring system: normal consistency feces were given a score of 0, semisolid feces were given a score of 1, and watery feces were given a score of 2. The sum of feces with scores of 1 and 2 was used for the analysis of the incidence of diarrhea.

Furthermore, at 1, 3, 7, and 14 dpi, fresh fecal samples (10 g) were collected from individual pigs for ST quantification. Fecal samples were serially diluted in buffered peptone solution (1:10) until they reached a final concentration of 10^−6^. From each dilution, 0.1-mL was plated on brilliant green agar containing Nal+ (25 μg/mL). Plates were incubated at 37 °C for 24 h. In the absence of bacterial growth on agar plates, an equal volume of Rappaport broth (CM0669; Oxoid, Thermo Fisher Scientific, Hampshire, UK), prepared at double concentration, was added to conic tubes containing the homogenized sample in PBS (1:10). The sample tubes were incubated at 37 °C for 24 h and plated again in Nal+ at 37 °C for 24 h. The number of colonies per gram of sample was transformed into log10 for further analysis.

Blood samples were taken at the jugular vein from all pigs at 0, 7, and 14 dpi. The pigs were fasted for six hours before blood sampling. For each sampling day, one 4-mL EDTA tube per animal was filled for blood cell count (Vacuplast; Cral, São Paulo, Brazil). Blood samples were stored on ice and transported to the laboratory, where the complete blood count was performed using a Microcell counter (Horiba Micros-60; Horiba ABX SAS, Montpellier, France).

### 2.4. Statistical Analysis

Data were tested for normality using the UNIVARIATE procedure of SAS (version 9.4, SAS Institute Inc., Cary, NC, USA) and the Shapiro–Wilk test. Studentized residuals were used to identify outliers (>3 standard deviations from the mean). Data were analyzed using the GLIMMIX procedure of SAS as a randomized complete design. The data collected over time were included in the analysis as a repeated effect, and each pig was considered the experimental unit. Differences between means were determined using the Tukey’s post-hoc test, except for the incidence of diarrhea. For this analysis, a Fisher’s exact test was used to compare treatments. The significance level adopted for all analyses was 5% (*p* < 0.05), and a trend toward significance was considered at *p* ≤ 0.10.

## 3. Results

### 3.1. Salmonella Typhimurium Fecal Shedding, Rectal Temperature, and Fecal Score

**Figure 1:**
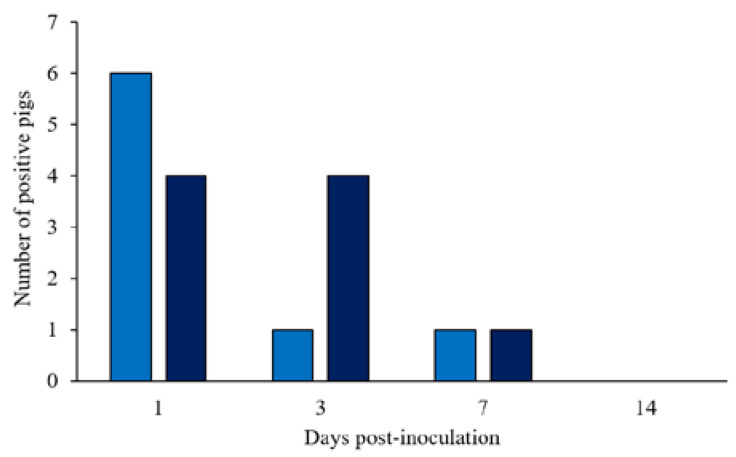
Number of positive pigs shedding fecal *Salmonella* Typhimurium after 1, 3, 7, and 14 days post-inoculation. (■) Pigs orally challenged with 1 × 10^8^ CFU of *Salmonella* Typhimurium; (■) Pigs orally challenged with 1.5 × 10^8^ CFU of *Salmonella* Typhimurium.

Pigs started shedding ST within 1 dpi (10 out of 20 pigs; Figure 1) and persistently shed the bacteria until the end of the first week of the study. The concentrations of *Salmonella* in the feces ranged from 2 to 6 log10 CFU/g of feces for the 1 × 10^8^ CFU group (6 out of 10 pigs) and from 2 to 4.5 log10 CFU/g feces for the 1.5 × 10^8^ CFU group (4 out of 10 pigs) at 1 dpi. The number of pigs shedding ST dropped markedly until 7 dpi (2 out of 20 pigs), however, pigs maintained higher colony counts, ranging from 4.7 to 6 log10 CFU/g. After 7 dpi, ST dropped in all samples, and it was not detectable on plates at 14 dpi. The Basal group remained negative until the end of the trial.

**Figure 2:**
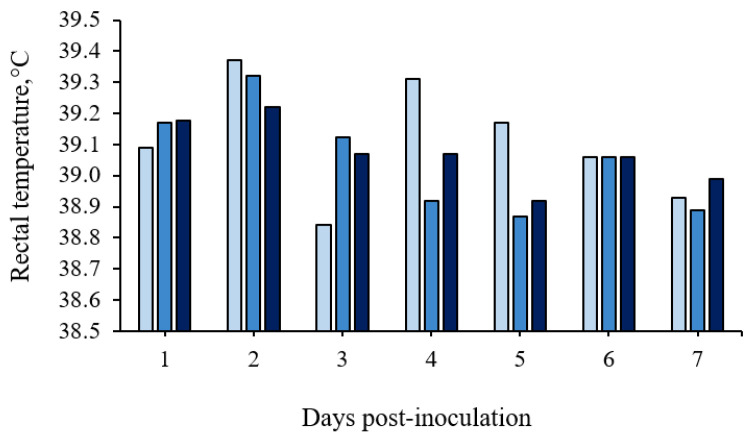
Rectal temperature means (°C) of growing pigs orally challenged or not with *Salmonella* Typhimurium. (■) Pigs without *Salmonella* Typhimurium (Basal); (■) Pigs orally challenged with 1 × 10^8^ CFU of *Salmonella* Typhimurium; (■) Pigs orally challenged with 1.5 × 10^8^ CFU of *Salmonella* Typhimurium. No differences between treatments were observed by the Tukey’s test (*p* > 0.05).

No significant differences were detected in rectal temperature between treatments (*p* > 0.10; Figure 2 and in details in Table S1 (Supplementary Materials)). However, infection with ST caused a febrile response in 35% of the ST-challenged gilts (7 out of 20 pigs; 3 pigs in the 1 × 10^8^ CFU group and 4 pigs in the 1.5 × 10^8^ CFU group), with rectal temperatures above 39.5 °C.

**Figure 3:**
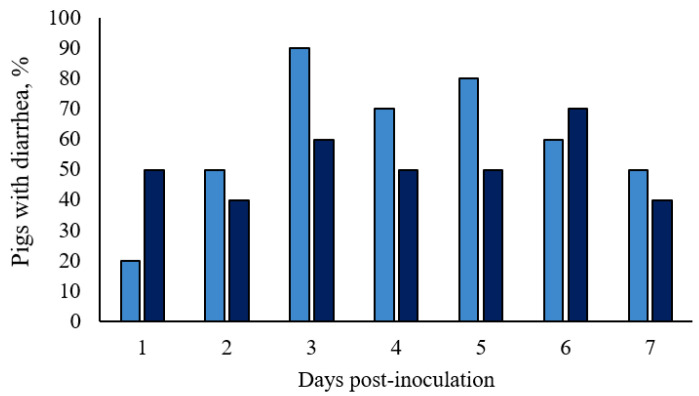
Incidence of diarrhea of growing pigs orally challenged with *Salmonella* Typhimurium. (■) Pigs orally challenged with 1 × 10^8^ CFU of *Salmonella* Typhimurium; (■) Pigs orally challenged with 1.5 × 10^8^ CFU of *Salmonella* Typhimurium. No differences between treatments were observed by Fisher’s exact test (*p* > 0.05).

In Basal pigs, normal fecal consistency (score 0) was observed in 70% (7 out of 10 pigs) of the animals from 1 to 4 dpi and in 80% of the animals from 5 to 7 dpi. Transitory diarrhea was observed in both ST-challenged groups, with semiliquid to liquid feces. Characteristic salmonellosis mild diarrhea (score 1) with the presence of mucus, started at 1 dpi, and a few episodes of severe diarrhea (score 2) occurred. Only one ST-challenged gilt in the 1 × 10^8^ CFU group scored two for 2 consecutive days, and one pig in the 1.5 × 10^8^ CFU group scored two for 1 day in the overall week. No significant differences were detected in fecal scores between treatments (*p* > 0.10; Figure 3 and in detail in Table S2), however, the 1.5 × 10^8^ CFU group had relatively stable percentages throughout the 7 days, while there were fluctuations among these percentages in the 1 × 10^8^ CFU group (Table S2). After a week of the challenge (7 dpi), half of the 1 × 10^8^ CFU pigs and 40% of the 1.5 × 10^8^ CFU pigs still had mild diarrhea.

### 3.2. Hematological Parameters

**Figure 4:**
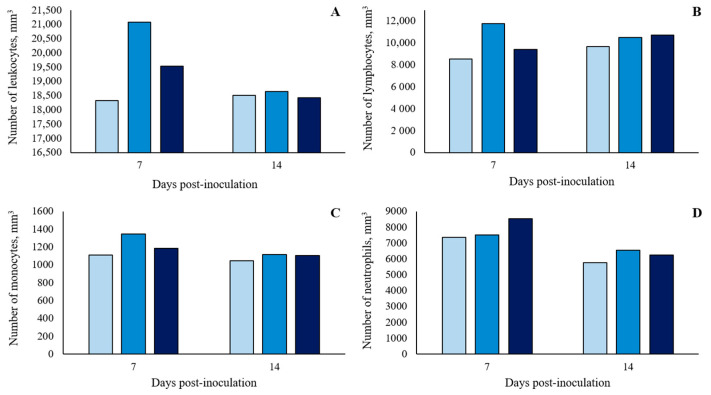
Number of leukocytes (**A**), lymphocytes (**B**), monocytes (**C**) and neutrophils (**D**) of growing pigs orally challenged or not with *Salmonella* Typhimurium. (■) Pigs without *Salmonella* Typhimurium challenge (Basal); (■) Pigs orally challenged with 1 × 10^8^ CFU of *Salmonella* Typhimurium; (■) Pigs orally challenged with 1.5 × 10^8^ CFU of *Salmonella* Typhimurium. No differences between treatments were observed by Tukey’s test (*p* > 0.05).

**Figure 5:**
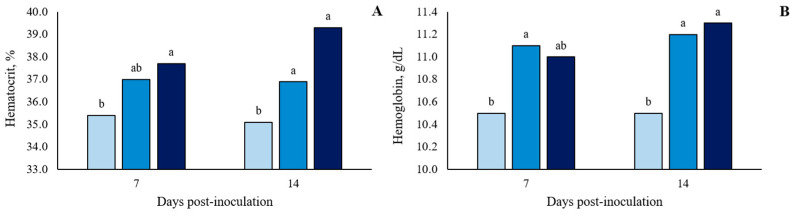
Hematocrit concentration (**A**) and hemoglobin content (**B**) of growing pigs orally challenged or not with *Salmonella* Typhimurium. (■) Pigs without *Salmonella* Typhimurium challenge (Basal); (■) Pigs orally challenged with 1 × 10^8^ CFU of *Salmonella* Typhimurium; (■) Pigs orally challenged with 1.5 × 10^8^ CFU of *Salmonella* Typhimurium. Means followed by the different letters on the same day differ by Tukey’s test (*p* < 0.05).

There was no significant (*p* > 0.10) treatment effect on the white blood cell (WBC) count at 7 and 14 dpi (Figure 4 and in detail in Table S3). However, there were two blood parameters that showed significant differences (*p* < 0.05) between treatments. For hemoglobin (HGB), at 7 and 14 dpi, oral ST-challenge increased HGB values in challenged pigs compared to Basal pigs (*p* < 0.05), with no significant difference between the dosages administered (*p* > 0.10; Figure 5 and in detail in Supplementary Table S3). In addition, the hematocrit (HTC) concentrations were affected by the ST-challenge. At 7 dpi, there was a greater HTC concentration in the 1 × 10^8^ CFU group than in the Basal group (*p* < 0.05), and no significant difference between the dosages administered (*p* > 0.10). On the other hand, at 14 dpi, the oral ST-challenge increased HTC values in both the 1 × 10^8^ and 1.5 × 10^8^ CFU groups compared to the Basal group (*p* < 0.05), with the 1.5 × 10^8^ CFU group showing a higher numeric HTC concentration than the 1 × 10^8^ CFU group (*p* > 0.10).

### 3.3. Growth Performance

All pigs were healthy and had similar growth performance in the pre-inoculation period (data not shown). In the first week of post-inoculation, there was no effect (*p* > 0.10) of ST challenge on BW and ADFI (Table 1). However, a steep reduction in ADG (−17 and −22%; *p* < 0.01) and G:F (−14 and −20%; *p* < 0.01) was observed in the first week post-inoculation in both ST-challenged groups compared to the Basal pigs, with no significant difference between the ST dosages (*p* > 0.10). However, for the experimental period (0 to 14 dpi), no significant differences were observed in BW, ADFI, ADG, and G:F between treatments (*p* > 0.10).

## 4. Discussion

The dose administration of 1 × 10^8^ and 1.5 × 10^8^ CFU in this study were chosen to more closely mimic the fecal–oral transmission of *Salmonella* organisms often found in intensive swine production conditions, as it was previously reported that growing–finishing phase pigs could shed high concentrations of ST in the environment (between 10^6^ and 10^8^ CFU/g of feces) [12,13]. Additionally, studies in which pigs were inoculated with doses between 10^6^ and 10^7^ showed the presence of mild infections or had no different outcomes from no-challenge animals [24,25]. Furthermore, when pigs were inoculated with higher doses of *Salmonella* Typhimurium (10^9^ CFU) severe clinical responses and mortality were observed [18]. Thus, choosing doses with 10^8^ CFU would activate the immune system of pigs without severe clinical symptoms or pig mortality. Moreover, the findings observed in this study suggest that both inoculum concentrations could produce a moderate immune response in pigs. The results also suggest that the biosecurity measures adopted in the current study were reasonably successful in preventing transmission of the ST infection to the Basal group (without *Salmonella* Typhimurium inoculation).

Studies performing the *Salmonella* Typhimurium challenge on weaning pigs often report the occurrence of clinical symptoms such as fever and yellowish watery diarrhea [19,26,27]. However, in this study the ST inoculation doses evaluated did not increase rectal temperature even with most animals excreting ST in the first week post-infection. The same results were reported by Davis et al. [25] in younger pigs inoculated with 10^8^ CFU of ST in a 14 days trial. The authors did not observe any differences in the rectal temperature of ST-inoculated pigs even when three doses were given at three different times.

The doses used in this trial may have been insufficient to overwhelm intestinal and mesenteric lymph node immunity, and thus, system migration and acute systemic inflammation may not have occurred. Indeed, Spiehs et al. [17] observed higher concentrations of ST in digestion related organs such as the ileum and the cecum than in the kidney, liver, and spleen when given an inoculum with 2 × 10^9^ CFU. This would also explain the lack of an effect on the WBC count observed in the current study. The low dosage of oral *Salmonella* Typhimurium used might have resulted in lower colonization of the mesenteric lymph nodes, spleen, and liver, which resulted in a mild and self-limiting immune response in the gut epithelium [28]. As a result, lower immune cell transmigration into the intestinal lumen might have occurred to avoid epithelial barrier disruption and pathogen translocation.

Although no differences were observed in WBC counts, blood parameters showed significant differences between treatments. HTC and HGB were greater in the 1.5 × 10^8^ CFU group than in the Basal and 1 × 10^8^ CFU groups. Increases in HTC and HGB concentrations could indicate the level of dehydration, since both indices are based on whole blood and are dependent on plasma volume. As challenged pigs showed an increased incidence of diarrhea after ST inoculation, the augmentation in HTC and HGB concentrations can be associated with fecal water loss. The presence of diarrhea is a characteristic sign of salmonellosis in pigs. This occurs due to the ability of *Salmonella enterica* serovar Typhimurium to induce the production of pro-inflammatory mediators in the gut barrier, altering chloride channel function and disrupting tight junctions, resulting in gut leakage and diarrhea [29].

Furthermore, higher HTC and HGB concentrations have been described as indicators of decreased performance [30] since both are related to impaired metabolism over the course of diarrhea observed in challenged pigs. A negative correlation between HTC and HGB and average weight gain in weaned piglets challenged with ST was observed [31]. It may occur because, under conditions of water restriction, growth may be impaired. First, the digestive processes of organic macronutrients (carbohydrates, proteins, and lipids) may have been compromised by the lower activity of hydrolase enzymes [32]. In addition, reduced water content is reported to inhibit the incorporation of amino acids into proteins [33], causing a decrease in protein accretion in the tissues. In this way, the increased HTC and HGB values observed at 7 and 14 dpi may partially explain the reduced growth performance (ADG) observed after 7 dpi.

Additionally, regarding growth performance, the gastroenteritis disorder caused by ST may have reduced nutrient digestion and absorption [34], lowering nutrient availability for growth. When pathogenic bacteria infect the intestinal epithelium, reduction in nutrient absorption and intestinal brush-border enzymes’ activities, and villous atrophy is induced by the inflammation caused by bacterial attachment and growth. Along with this, crypt hyperplasia, due to increased stem cell proliferation in the crypt, is promoted for the regeneration of intestinal enterocytes [3]. Thus, gut barrier disruption may explain the impact of ST on growth performance observed in the first week. In addition, the absence of ADFI reduction in both ST groups observed in this study confirms that ST affects pigs’ performance indirectly via nutrient utilization efficiency rather than via a reduction in voluntary feed intake. Meanwhile, 1.5 × 10^8^ CFU pigs had a more pronounced reduction in ADG and G:F when compared to the Basal group, which can be related to the higher dose concentration.

However, a negative effect on performance from 0 to 14 dpi was observed in either of the ST-challenged groups. The lack of effect on growth parameters might be associated with the inoculating concentration used in the trial where only 10% of the pigs (2 out of 20 pigs) were shedding ST at 7 dpi, and all inoculated pigs were negative at 14 dpi. Moreover, unlike field conditions, in this trial, pigs were housed individually in pens with fully slatted floors. This housing system prevented challenged pigs from being re-infected by the fecal–oral cycle, especially long-term carrier pigs that can, continuously or intermittently, shed ST in feces as a way of environmental contamination [9]. These results are similar to those observed by Davis et al. [24], who did not notice differences in the BW and ADFI of piglets inoculated with three doses of ST. Additionally, they demonstrated that the serum IGF concentration was reduced when *Salmonella* Choleraesuis was inoculated, but no differences were noticed in piglets inoculated with *Salmonella* Typhimurium. The authors hypothesize that the lack of difference between the control and ST-inoculated group occurred due to low ST pathogenicity, which may not cause a systemic immune activation capable of impairing growth.

Taken together, the results demonstrate that both concentrations of the oral ST challenge model were able to affect pig performance and blood parameters. However, the oral dosage of 1.5 × 10^8^ CFU of *Salmonella* Typhimurium is a more suitable dosage to activate the immune system of pigs and impair growth performance.

## Figures and Tables

**Table 1:** Growth performance of growing pigs orally challenged or not with *Salmonella* Typhimurium.

Item	Inoculation Level, CFU			SEM	*p*-Value
	0 (Basal)	1 × 10^8^	1.5 × 10^8^		
BW, 0 dpi	27.25	27.23	27.42	1.66	0.99
BW, 7 dpi	32.95	31.94	31.90	1.80	0.89
BW, 14 dpi	38.10	37.79	37.74	2.04	0.99
0 to 7 dpi					
ADG, kg	0.81 ^a^	0.67 ^b^	0.64 ^b^	0.03	<0.01
ADFI, kg	1.55	1.52	1.54	0.07	0.95
G:F, kg/kg	0.52 ^a^	0.45 ^b^	0.42 ^b^	0.02	<0.01
0 to 14 dpi					
ADG, kg	0.84	0.75	0.74	0.04	0.26
ADFI, kg	1.66	1.63	1.68	0.07	0.86
G:F, kg/kg	0.46	0.47	0.44	0.01	0.22

SEM, Standard error of the mean. ^a,b^ Within a row, means not sharing the same superscript letter differ, *p* < 0.05.

## Data Availability

Due to our institutional policy, data will be available upon request only.

## Supplementary Materials

The following supporting information can be downloaded at: https://www.mdpi.com/article/10.3390/microorganisms11020446/s1, Table S1: Rectal temperatures (°C) of growing pigs orally challenged or not with *Salmonella* Typhimurium.; Table S2: Incidence of diarrhea after *Salmonella* Typhimurium inoculation.; Table S3: Blood parameters in growing pigs orally challenged or not with *Salmonella* Typhimurium.
