# Supplementary material for: A Simple Assay to Assess Salmonella Typhimurium Impact on Performance and Immune Status of Growing Pigs after Different Inoculation Doses
Source: Microorganisms. 2023 Feb 10;11(2):446. doi: 10.3390/microorganisms11020446 (PMC9962513; doi:10.3390/microorganisms11020446)
Supplement: Supplementary file 1 [file microorganisms-11-00446-s001.zip › microorganisms-2182300-supplementary.pdf]

Table S1. Rectal temperatures (°C) of growing pigs orally challenged or not with *Salmonella* Typhimurium.

Days post-inoculation	Inoculation level, CFU			SEM	*P*-value
	0 (Basal)	1×10^8	1.5×10^8		
1	39.1	39.2	39.2	0.05	0.71
2	39.2	39.3	39.2	0.05	0.58
3	38.9	39.0	39.0	0.06	0.17
4	39.2	39.0	39.1	0.05	0.11
5	39.1	38.9	38.9	0.04	0.11
6	39.1	39.1	39.1	0.04	0.99
7	38.9	38.9	39.0	0.05	0.80

SEM, Standard error of the mean.

Table S2. Incidence of diarrhea[1] after *Salmonella* Typhimurium inoculation.

Days post-inoculation	Inoculation level, CFU		*P*-value
	1×10^8	1.5×10^8	
1	20%	50%	0.13
2	50%	40%	0.49
3	90%	60%	0.28
4	70%	50%	0.32
5	80%	50%	0.24
6	60%	70%	0.67
7	50%	40%	0.49

[1]The percentage of diarrhea was calculated as a proportion of the total number of pigs showing feces scored as 1 and 2 per treatment from 1 to 7 dpi.

Table S3. Blood parameters in growing pigs orally challenged or not with *Salmonella* Typhimurium.

Item	Inoculation level, CFU			SEM	*P*-value
	0 (Basal)	1×10^8	1.5×10^8		
Leucocytes, mm^3					
7 dpi	18,320	21,080	19,530	1683	0.37
14 dpi	18,510	18,650	18,430	1273	0.73
Lymphocytes, mm^3					
7 dpi	8,543	11,788	9,427	1360	0.39
14 dpi	9,658	10,490	10,732	727	0.73
Monocytes, mm^3					
7 dpi	1,110	1,345	1,186	133	0.39
14 dpi	1,045	1,115	1,103	78	0.96
Neutrophils, mm^3					
7 dpi	7,368	7,507	8,551	760	0.85
14 dpi	5,758	6,535	6,256	565	0.33
Hematocrit, %					
7 dpi	35.4[b]	37.0[ab]	37.7[a]	0.64	0.03
14 dpi	35.1[b]	36.9[a]	39.3[a]	0.68	<0.05
Hemoglobin, g/dL					
7 dpi	10.5[b]	11.1[a]	11.0[ab]	0.18	0.05
14 dpi	10.5[b]	11.2[a]	11.3[a]	0.20	<0.01

SEM, Standard error of the mean.
[a,b] Within a row, means not sharing the same superscript letter differ, $p < 0.05$.
